# The real experience and coping styles of emergence delirium in patients after radical lung cancer surgery: a qualitative study

**DOI:** 10.3389/fpsyt.2026.1790981

**Published:** 2026-09-28

**Authors:** Tongyang You, Qin Li, Yao Fu, Juan Wang

**Affiliations:** The Affiliated Hospital of Southwest Medical University, Luzhou, China

**Keywords:** emergence delirium, lung cancer, qualitative study, radical lung cancer surgery, real experience

## Abstract

**Background:**

Emergence delirium (ED) is a prevalent and complex complication in the post-anesthesia care unit (PACU) that significantly impacts patients undergoing radical lung cancer surgery. Despite its high incidence, the subjective experience of ED, along with patients’ coping strategies and support needs, remains insufficiently understood. This study aims to address this gap by exploring the real experiences of patients who develop ED following radical lung cancer surgery.

**Methods:**

To gain an in-depth understanding of the real experience of emergence delirium in patients after radical lung cancer surgery, a qualitative descriptive phenomenological study was conducted at a tertiary hospital in Sichuan, China. Semi-structured in-depth interviews were performed with 15 patients who experienced ED after radical lung cancer surgery. Data were analyzed using Colaizzi’s seven-step method to extract significant statements and formulate themes.

**Results:**

Participants described ED as a distressing experience characterized by intense perceptual disturbances, including audiovisual hallucinations, temporal-spatial and situational disorientation, and uncontrollable behavioral abnormalities. Participants described complex emotional responses during the episode: most reported intense fear, anxiety, and anger during the acute phase, followed by persistent feelings of shame and guilt after the episode, while a minority exhibited neutral or even euphoric moods. Coping patterns diverged: some chose to avoid and forget the experience, while others actively sought to understand its causes. Furthermore, ED episodes resulted in significant lasting consequences, including varying degrees of memory impairment, damaged self-esteem, and fear of recurrence. Finally, participants consistently expressed unmet support needs, primarily family companionship, psychological counseling, and clear explanations about ED from healthcare providers.

**Conclusion:**

ED is experienced by patients as a complex event marked by intense perceptual disturbances, loss of bodily control, and profound negative emotions including fear and shame. Patients mobilize both avoidant and positive meaning-seeking coping strategies. These findings underscore the urgent need for systematic ED screening, acute-phase acceptance-based bedside care, clear post-episode communication, family involvement, and individualized follow-up to support comprehensive psychological and physical recovery.

## Introduction

Lung cancer has the highest mortality and morbidity rates of all malignant tumors in China (1). Currently, surgery-based comprehensive treatment is the preferred strategy for lung cancer management (2). In recent years, thoracoscopic radical resection has gained widespread clinical popularity, proving to be a reliable procedure for lung tumor removal (3). Although characterized by minimal invasiveness and relatively minor trauma, it is prone to postoperative neuropsychiatric complications, with delirium being one of the most common (4, 5), occurring at an incidence of 5-16% (6). Delirium is a frequent postoperative complication, particularly among elderly patients, often associated with adverse clinical outcomes that severely impact prognosis (7).

Based on the time of onset, delirium can be categorized into emergence delirium (ED) and postoperative delirium (POD) (8). ED is an acute cognitive dysfunction occurring from the immediate cessation of anesthesia to the early post-anesthesia period, predominantly manifesting in the post-anesthesia care unit (PACU). It represents an acute neuropsychiatric syndrome characterized primarily by impairments in attention and cognitive function. ED is classified into three subtypes: hypoactive, hyperactive, and mixed, and is most frequently observed in elderly and pediatric populations (9). Clinically, it presents with acute onset, marked by hyperactive and agitated altered mental states, alongside acute confusion. Studies have demonstrated that the occurrence of ED in the PACU is a causal factor for an increased risk of POD, long-term cognitive decline, and prolonged hospitalization (9). Currently, research on delirium predominantly focuses on POD, with comparatively less attention directed toward ED, and the majority of these studies are quantitative. Therefore, this study aims to explore the real experiences of ED in patients following radical lung cancer surgery, providing a reference for healthcare professionals to accurately identify and manage ED, ultimately reducing the incidence of POD and improving patient outcomes.

## Materials and methods

### Aim

To explore the real experiences of ED in patients following radical lung cancer surgery, identify their coping strategies, and provide a basis for targeted, personalized nursing interventions to improve the patient experience.

### Design

A descriptive phenomenological qualitative approach was utilized. Data were collected through face-to-face, one-on-one semi-structured interviews. The interview guide was initially formulated based on the study objectives, literature review, and group discussions. Following pilot interviews with two ED patients, the guide was refined for understandability and acceptability, and finalized after consultation with six experts. Using the opening question, “Can you talk about your experience after surgery?” to build rapport, the guide included: 1) Did you see or hear anything after surgery that others did not? Can you describe it? 2) Do you know where you were located and what you were doing? 3) What kind of emotions were you feeling? 4) What kind of help would you have liked to have received? 5) How did this experience affect you physically and psychologically? 6) How did you cope with this experience? 7) Is there any kind of support or help you would like to receive at this time?

### Data collection and participants

Purposeful sampling was employed to recruit patients who experienced ED after radical lung cancer surgery at a tertiary hospital in Sichuan Province between January and September 2024. The Nu-DESC scale assessment was performed exclusively by uniformly trained PACU nurses, initiated immediately after patients regained consciousness and were successfully extubated, and repeated every 10 minutes until discharge. Each item is scored on a 3-point scale (0-2), with a maximum total score of 10. A total score of ≥1 (10) has been established as the validated cutoff score for ED detection (11). A definitive diagnosis of ED was confirmed only when both a Nu-DESC score ≥1 and an independent positive clinical evaluation by an attending anesthesiologist in the PACU were met. Semi-structured interviews were conducted on the 3rd postoperative day.

The inclusion criteria were as follows: (1)age ≥18 years; (2)underwent thoracoscopic radical surgery (anatomical lobectomy or sublobar resection, including anatomical segmentectomy and radical wedge resection);(3) confirmed ED during the PACU stay; (4) clear consciousness and adequate communication skills on postoperative day 3; (5) provided informed consent.

Exclusion criteria: (1) history of psychiatric illness or cognitive impairment; (2) hearing or speech disorders; (3) withdrawal midway; (4) persistent delirium on postoperative day 3. Sample size was determined by data saturation (12), which was reached at the 15th participant, where no new themes emerged, prompting the cessation of data collection.

### Ethical considerations

Prior to the interviews, researchers explained the purpose and content to the patients, assuring them that their real names would be replaced by admission numbers to protect their privacy, and promising strict confidentiality throughout the process. Upon obtaining consent, the interviews were audio-recorded in their entirety. All participants signed informed consent forms in accordance with the Declaration of Helsinki. To protect privacy, participants were numbered P1 to P15. This study was approved by the Ethics Committee of the Affiliated Hospital of Southwest Medical University (Approval No. KY2024439).

### Data collection methods

To minimize interviewer bias and ensure consistency, all interviews were conducted by the same two trained researchers throughout the study. One researcher assumed the role of primary interviewer, responsible for guiding the semi-structured conversation, while the other acted as a non-participant observer and recorder. Both researchers completed a 20-hour specialized training program in qualitative research methods, including phenomenological interviewing skills, active listening techniques, and reflexive practice, prior to initiating data collection. Interviews were held in a dedicated, soundproof consultation room in the Thoracic Surgery outpatient clinic to ensure privacy and minimize interruptions. Before each interview, informed consent was reconfirmed, and a warm-up conversation was conducted to build rapport. The primary interviewer’s proficiency in both Mandarin and the local Sichuan dialect facilitated authentic communication and prevented misinterpretation of nuanced emotional expressions. The recorder maintained detailed field notes throughout each interview, capturing non-verbal behaviors, pauses, emotional shifts, and contextual details that would not be evident in audio recordings alone. All field notes were cross-referenced with verbatim transcripts within 24 hours of each interview. Interview durations ranged from 28 to 61 minutes, with a mean of 40 minutes.

### Data analysis

Within 24 hours after the interview, the primary interviewer repeatedly listened to the recordings and transcribed them verbatim into standard written Chinese, fully preserving the contextual meaning of the dialect. The transcripts were checked against field notes and verified with the participants to ensure authenticity; another researcher cross-checked the transcribed information. Colaizzi’s 7-step analysis (13) method was used to Analyze the data: (1)Thoroughly read the material to fully understand the content; (2) Identify meaningful statements or descriptive sentences;(3) Organize meaningful statements into units of meaning; (4) Categorize and induct units of meaning to identify common themes; (5) Provide detailed descriptions of each theme, summarizing its characteristics and essence; (6) Derive a fundamental structure from the detailed descriptions, clarifying core content; and (7) Validate the accuracy and completeness of the fundamental structure through feedback. Two trained researchers independently coded the data using Microsoft Word to identify significant statements, formulate meanings, and cluster them into themes. Disagreements were resolved through group discussion to reach a consensus. The study strictly adhered to the COREQ guidelines for qualitative reporting to ensure transparency and rigor.

### Researcher reflexivity and positionality

According to Husserl’s phenomenological reduction theory (14), researchers must temporarily suspend (bracket) their everyday presuppositions and theoretical frameworks to achieve a pure description of the phenomenon. The primary interviewer is a Master of Nursing and a charge nurse with 3 years of operating room and 2 years of PACU experience, possessing extensive knowledge of ED and perioperative delirium. Prior assumptions included the belief that ED patients experience negative emotions like fear and anxiety and have high demands for medical support. To control the influence of these assumptions, the interviewer kept a reflexive journal before each interview. To avoid prior contact with participants, the interviewer applied to work in a different campus of the hospital (without thoracic surgery patients) during the study period. Phenomenological questioning techniques (e.g., “Please describe the most unique feeling of this experience”) and contextual tracing (placing meaning units back into the full interview context) were used to maintain bracketing. Throughout the process, a neutral stance was maintained, utilizing open-ended questions and avoiding leading language.

## Results

In total, 15 patients participated (6 females, 9 males), with a mean age of 64.47 ± 9.71 years (range: 43–84 years). Regarding marital status, 12 were married and 3 were widowed. Educational attainment varied: 2 illiterate, 6 primary school, 2 junior high school, 3 senior high school, and 2 secondary technical school. Detailed patient information is presented in **Table 1**. Five main themes and fifteen subthemes were identified (**Table 2**).

**Table 1:** Basic patient information.

Number	Sex	Age	Marital status	Surgical procedures	Types of ED
P1	female	65-70	Married	Anatomical lobectomy	Hypoactive
P2	male	70-75	widowed	Anatomical lobectomy	Mixed
P3	male	65-70	Married	Anatomical lobectomy	Hyperactive
P4	male	70-75	Married	Anatomical lobectomy	Hypoactive
P5	male	60-65	Married	sublobar resection^a^	Hyperactive
P6	male	50-55	Married	sublobar resection^b^	Hypoactive
P7	female	65-70	Married	Anatomical lobectomy	Hyperactive
P8	male	55-60	Married	Anatomical lobectomy	Hyperactive
P9	male	70-75	widowed	sublobar resection^b^	Hypoactive
P10	male	70-75	Married	Anatomical lobectomy	Mixed
P11	male	80-85	widowed	sublobar resection^b^	Hypoactive
P12	female	60-65	Married	Anatomical lobectomy	Hypoactive
P13	female	50-55	Married	sublobar resection^b^	Mixed
P14	female	60-65	Married	Anatomical lobectomy	Hyperactive
P15	female	40-45	Married	Anatomical lobectomy	Hyperactive

a: radical wedge resection; b: anatomical segmentectomy.

**Table 2:** Themes and subthemes of the study.

Theme	Subtheme	Main empirical evidence	Interpretive meaning	Implication for care
Theme 1: Profound Experience of Perceptual Disturbances During ED	1.1 Positive and negative audiovisual hallucinations	“I … saw a lot of faces floating in the air with no bodies…” (P1); “I heard a voice … to die.” (P14); “I heard deafening music” (P11); “I had a good hand… (laughs)” (P4)	**Erosion of Bodily and Cognitive Autonomy**: Patients experienced hallucinations (auditory and visual) and disorientation in time, space, and situation, along with expressive disorders, further underscoring the disintegration of bodily and cognitive autonomy. When the brain cannot distinguish reality from dreams and cannot anchor itself in the current time, physical space, and situation, the patient’s coherent self-awareness becomes fragmented; coupled with the inability to control the body (inability to execute purposeful actions or express needs), patients descend into deeper panic, generating intense outward emotions (e.g., anger, anxiety) and external behavioral abnormalities (e.g., vigorous physical struggles).	**Do not invalidate patients’ perceived reality:** Avoid directly contradicting patients’ hallucinations (visual and auditory); gently provide reality orientation, soothe emotions, and use physical restraint with caution.
	1.2 Temporal and spatial disorientation	“I didn’t know where I was … I thought I was back in my 50’s…” (P2); “I thought that time had stood still…” (P6)		**Continuous environmental reorientation:** Provide simple, consistent reality cues (e.g., clocks, calendars, familiar objects), repeatedly inform patients of the time, place, and situation, and help rebuild a sense of security.
	1.3 Behavioral abnormalities and expressive disorders	“I have no way of answering him or controlling my body…” (P3); “I was not in control of myself at all…” (P8)		**Use physical restraint with caution:** If restraint is necessary, explain in advance, maintain patience, and provide reassuring physical contact.
	1.4 Situational disorientation	“I thought that I was planting rice seedlings in the field…” (P10); “I felt I was floating in a white field…” (P9)		Accept feelings first, then gently correct; do not force arguments.
Theme 2: Positive and Negative Emotions in the Face of ED	2.1 Fear and anxiety	“Going to kill me and eat my flesh” (P11); “I didn’t have any control over my hands … very anxious and upset” (P13)	**Existential Threat and Helplessness:** Fear and anxiety stem from existential threat and helplessness. Patients’ hallucinations were interpreted as direct threats to life, while the loss of behavioral self-control deprived them of their basic sense of security, both triggering intense survival instincts.	**Calm companionship and reassurance:** Healthcare providers should remain calm, use soothing language and physical contact (e.g., holding hands), help patients feel safe, and avoid confrontation or expressions of impatience.
	2.2 Anger and a sense of not being understood	“I was so annoyed that I wanted to stand up…” (P3); “…wanted to get rid of the person who was pressing on my legs … I was angry” (P7)	**Frustration of Autonomy:** Anger is an instinctive response to the frustration of autonomy and the invalidation of one’s perceived reality. When healthcare providers or family members cannot understand the patient’s distorted sense of reality and restrict their instinctive movements, this exacerbates their anger.	
	2.3 Shame and guilt	“I couldn’t help it I felt guilty.” (P8); “I was embarrassed and to this day I feel ashamed.” (P14)	**Threat to Social and Family Identity:** The loss of behavioral control during ED episodes is profoundly at odds with patients’ established family and social roles (e.g., teacher, elder), leading to damaged dignity and a lasting psychological burden.	**Normalization and psychological repair:** Clearly inform patients that ED is a common complication of anesthesia, not a personal failing or mental illness, to alleviate their moral burden; refer for psychological counseling when necessary.
	2.4 Neutrality and euphoria	“I didn’t feel anything at the time” (P4); “I thought I was at home with my grandchildren and had no (mood) swings.” (P9)	**Potential Cognitive Impairment:** Even neutral or pleasant emotions cannot conceal the essential erosion of cognitive autonomy. Patients are completely detached from present reality; although this disconnection is temporarily experienced as benign, it underscores the rupture between the self and reality, carrying equivalent potential risks of cognitive impairment.	**Nursing alert for benign-appearing presentations:** Calm appearances may mask cognitive impairment. Do not be misled by benign emotions. Continuous consciousness assessment is needed to prevent adverse events.
Theme 3: Diverse Coping Strategies for Managing ED	3.1 Negative coping	“I don’t want to think back on what happened then…” (P1); “I want to forget this bad experience” (P6)	**Need to Reconstruct Meaning:** Negative coping reflects avoidance of psychological trauma associated with the loss of autonomy, which may lead to long-term medical fear; positive coping represents active cognitive efforts to rationalize bizarre behaviors by seeking medical explanations, reintegrating the chaotic ED episode into a coherent personal narrative, thereby restoring predictability, control, and psychological equilibrium.	**Respect the patient’s pace; do not force recall:** Before the patient is ready, do not compel them to recall or discuss details; provide written information for later review to reduce uncertainty.
	3.2 Positive coping	“I’m looking for the reasons why it happened to me…” (P15); “I probably miss that time so much that I did that” (P10)		**Clear health education:** Proactively provide clear explanations about ED (causes, mechanisms, prognosis), answer patients’ questions about “why this happened,” and help rebuild trust in their body and in healthcare.
Theme 4: Outcomes of Experiencing ED	4.1 Variable abilities to recall the ED experience	“I can’t remember at all what happened” (P12); “I don’t know how long I was like this” (P6); “My memory has become very bad” (P7)	**Biographical Disruption:** The enduring consequences of ED indicate that the erosion of bodily and cognitive autonomy and the threat to social identity do not dissipate immediately upon recovery of consciousness. Memory gaps and the awareness of bizarre behaviors learned only through others’ accounts create a “biographical disruption,” forcing patients to confront altered self-perceptions and generating anticipatory anxiety.	**Sustained attention and support:** Acknowledge patients’ sense of loss, provide ongoing psychological support, help patients integrate this “missing” experience, and repair their damaged self-image.
	4.2 Damaged self-esteem and long-term shame	“I couldn’t listen to anyone’s advice … I feel so ashamed just thinking about it.” (P8); “I was always under a lot of pressure” (P15)		Provide sustained psychological support, normalize the delirium experience, and refer for professional psychological counseling when necessary.
	4.3 Fear of recurrence and concern about disease prognosis	“I don’t dare to go to the hospital” (P11); “I’m more worried that this experience is going to make my disease worse” (P13)		**Prognostic reassurance and follow-up:** Clearly inform patients that ED is typically transient and does not directly affect lung cancer prognosis; provide security through discharge follow-up (telephone/Internet) to reduce avoidance behaviors.
Theme 5: Healthcare Management Needs in the Face of ED	5.1 Desire for family companionship and psychological support	“I wished that a family member would stay with me, and talk to me.” (P3); “I didn’t want to face that fear alone.” (P1)	**Attachment Need for Loved Ones:** This desire reflects patients’ attachment needs for loved ones and close friends in a vulnerable state. Family members and close friends serve as anchors of reality and sources of safety; their companionship can provide a familiar connection when patients’ self-awareness is fragmented, helping to rebuild a sense of security.	**Family-participatory care:** Where PACU conditions permit, create opportunities for family visits or “cloud companionship”; integrate psychological support into routine care pathways.
	5.2 Weakness of the support system and desire for clear explanations and continued attention	“I hope the doctor can give me a clear explanation.” (P12); “I would like to have a doctor who keeps an eye on my condition” (P13)	**Need to Reduce Uncertainty:** Patients’ strong desire for clear explanations and continued medical attention fundamentally stems from the need to reconstruct meaning after a confusional experience and to alleviate threats to identity and health. Authoritative and empathetic explanations from healthcare providers not only serve an educational function but also validate patients’ experiences, reassure them that their cognitive autonomy remains intact, and thereby rebuild their trust in the healthcare system and their own bodily integrity.	**Standardized explanations and continuity of care**: Develop standardized ED educational materials; leverage “Internet + Nursing” to provide post-discharge continuity of care, meeting patients’ needs for ongoing attention.

Bold text indicates the interpretive meaning and implication for care as synthesized summaries for each subtheme.

### Theme 1: profound experience of perceptual disorders in the face of ED

Subtheme 1.1 positive and negative audio-visual hallucinations: Respondents recalled their post-surgical experiences, most of which were perceived as unreal experiences or psychotic dreams or experiences of living in another world. A portion described unreal images they were seeing and voices they were hearing, unable to distinguish between reality and hallucinations. The types of visual hallucinations ranged from concrete creature types to abstract forms and geometric shapes. Auditory hallucinations ranged from concrete sounds, such as rain, music, and conversations, to indistinct voices.

“I recall now that I was dreaming like I was lying on a very small bed and saw a lot of faces floating in the air with no bodies, and I heard a lot of babies crying and it was scary.” (P1)

“I remember it a little bit blurry, I just remember I kept trying to urinate and I was very uncomfortable … it was pouring rain outside and I tried to run to a place where there was no one to go to the toilet, but I always saw a bunch of people running after me, they were laughing and talking to each other about something, I couldn’t hear them clearly, and I saw a lot of twisted and turned squares in front of me blocking me.”(P5)

“I saw butterflies next to me giving me fluids in a variety of fruits.” (P13)

Some interviewees mentioned feeling that their lives were threatened at the time.

“I was tied up and the ceiling was all spinning, and I heard deafening music, and the doctor was going to kill me, and I thought I was going to die there.” (P11)

“I just heard a voice that kept telling me to die. I just threw up my fists and tried to beat the voice away.”(P14)

A small minority of participants also experienced positive audio-visual hallucinations that led to involuntary behaviors.

“I thought I was playing mahjong … I had a good hand and was about to win three houses (laughs), and I kept touching the cards.”(P4)

Analytical interpretation: Unable to distinguish reality from dreams, patients were trapped in an inescapable non-reality. This loss of cognitive control triggered primitive survival instincts, explaining the intense physical struggling observed by staff.

Subtheme 1.2 temporal and spatial disorientation: Participants experienced a state of temporal and spatial confusion throughout the ED. They were unable to determine their location, often perceiving themselves to be in multiple places simultaneously, and were either unaware of the current time or found it confusing, resulting frequently in agitation.

“I was simply forgetting that I was having the operation, and I didn’t know where I was … I thought I was back in my 50’s…”(P2).

“I was so disorganized, I knew I was in the hospital but I felt like I was at home, it was like I was splitting into two people … I just kept trying so hard to figure out the world and it probably came across as extreme.” (P7).

Some interviewees also mentioned that they did not feel the passage of time during the ED and thought that time was at a standstill.

“I thought that time had stood still, and I saw that the clock on the wall hadn’t moved either…”(P6).

Analytical interpretation: Temporal-spatial disorientation further highlights the destruction of cognitive autonomy. When patients cannot anchor the “self” in current time and physical space, coherent self-consciousness fragments.

Subtheme 1.3 behavioral anomalies and expressive disorders: Interviewees mentioned that they were unable to control their demeanor or unconsciously performed some abnormal actions, which were different from the patient’s daily life, and they were unable to act according to instructions. Patients also often felt unable to communicate and express their needs, and their language was not logical.

“I hear someone asking me questions, asking my name, telling me to stick my tongue out, talking to me all the time, but I have no way of answering him or controlling my body, and I just keep trying to get up.” (P3)

“I always felt like there was something in the right side of my chest that kept pushing against me very hard, and then they told me that I kept trying to pull on the closed chest drain at that point. The truth is that in the moment I was not in control of myself at all, I could only follow my instincts, I didn’t want to be uncomfortable … and I didn’t mean to be.”(P8)

Analytical interpretation: This reflects a severe deprivation of bodily autonomy. The inability to execute purposeful movements or express needs led to intense internal frustration, externalizing as behavioral anomalies.

Subtheme 1.4 situational disorientation: patients mentioned being conscious, believing themselves to be in another situation or jumping back and forth between multiple situations, and not being able to think clearly.

“I don’t actually remember too much, I thought that I was planting rice seedlings in the field, and I saw my old partner, who had already been there, also with me. “(P10)

“One moment I felt I was floating in a white field, then I heard my grandchildren’s voices and thought I was with them.”(P9)

Analytical interpretation: Situational disorientation embodies the dual destruction of bodily and cognitive autonomy. Unable to integrate the current reality, consciousness regressed or escaped into fragmented illusions, exacerbating internal chaos.

### Theme 2: positive and negative emotions in the face of ED

Subtheme 2.1 fear and anxiety: Some patients feel fear and anxiety when in the face of ED, this may be related to patients experiencing negative audiovisual hallucinations of varying intensities, believing that their lives were threatened.

“It’s very scary, it’s like a dream it’s like a dream, I want to wake up but I can’t wake up I feel so tired.” (P1).

“I always feel like someone is going to kill me and eat my flesh, I’m terrified even now thinking about it.” (P11).

Loss of control and autonomy of the patient’s behavior and excessive differences from everyday behavior are also common causes of fear and anxiety.

“I heard someone keep calling my name … my son gave me the story that he was calling me and I couldn’t respond to him and I didn’t have any control over my hands to catch the butterflies, I was feeling very anxious and upset. “ (P13).

“I thought I was crazy. I was very grumpy at the time, nothing like my usual good nature. I was panicking inside.”(P15).

Analytical interpretation: Fear and anxiety stem from existential threat and helplessness. Hallucinations were interpreted as direct threats to life, while loss of self-control stripped away basic safety, triggering strong survival instincts.

Subtheme 2.2 anger and a sense of not being understood: Some patients exhibited extreme agitation and anger when faced with ED, experienced imaginary scenarios, and were incapable of differentiating between hallucinations and reality. They perceived that medical staff and family members failed to comprehend their experiences and refused to acknowledge their legitimate needs, including defecation and standing up.

“I was so annoyed that I wanted to stand up and breathe heavily, but the next to me nurse wouldn’t allow me to do that, so I got angry and struggled vigorously, and then my hands were tied up and I had a feeling of abandonment and not being understood.” (P3).

“I didn’t even know what world I was in, I was confused also fighting with myself, I wanted to get rid of the person who was pressing on my legs and grabbing my things … I was angry.” (P7).

Negative work attitudes of medical staff can also make patients in ED feel angry and suspicious and distrustful of medical staff.

“I see the nurses being impatient with me, not taking me seriously and even laughing at me, which makes me even more angry and makes me feel that they are not going to treat me properly.”(P6).

Analytical interpretation: Anger is an instinctive response to the frustration of autonomy and the invalidation of one’s perceived reality. When healthcare providers or family members are unable to understand the patient’s distorted sense of reality and restrict their instinctive movements, this exacerbates their anger.

Subtheme 2.3 shame and guilt: Some participants mentioned experiencing feelings of shame, embarrassment, and guilt toward medical staff and family members during ED, which persisted for some time after the end of ED, and in some cases, permanently.

“I was trying to go and pull the tubes off me, I didn’t want to feel bad, I knew they were all doing it for my own good, I couldn’t help it I felt guilty. “ (P8).

“I don’t remember much … I was listening to my daughter later telling me about some of my behavior at the time and I was embarrassed and to this day I feel ashamed.”(P14).

Analytical interpretation: ED poses a severe threat to patients’ social and family identity. The loss of behavioral control during ED episodes is profoundly at odds with patients’ established family and social roles (e.g., teacher, elder), leading to a sense of damaged dignity. Their suffering stems not only from the illness itself but also from the perceived destruction of their image in the eyes of others.

Subtheme 2.4 neutrality and euphoria: Patients experiencing positive audio-visual hallucinations during episodes of ED exhibited either a neutral mood or no discomfort, and, in some cases, exhibited agitation and euphoria.

“I didn’t feel anything at the time, I was very much at home playing cards with my friends.” (P4).

“I heard my grandchildren calling out to me, I thought I was at home with my grandchildren and had no (mood) swings.”(P9).

Patients may feel agitated if they think they have a strong power or that the experience is unreal.

“I was thinking that I had gained a superpower and was able to fly in the sky, and I saw my partner whom I hadn’t seen for a long time and I knew in my heart that it was unreal, so I didn’t have a burden on my mind … and felt excited, I missed her. “(P10).

Analytical interpretation: Even neutral or pleasant emotions cannot conceal the essential erosion of cognitive autonomy. Patients are detached from present reality; although this disconnection is temporarily experienced as benign, it in fact underscores the rupture between the self and reality, carrying equivalent potential risks of cognitive impairment.

### Theme 3: various strategies for managing ED

Subtheme 3.1 negative coping: The patient will try to forget the event by avoiding talking about it with others or recalling it by adopting silence, avoidance, or changing the topic of conversation about some specific experience of ED.

“I don’t want to think back on what happened then, it’s all in the past. That dream was too tiring.” (P1).

“I can’t remember… (Silence) My brain turns on like a defense mechanism, he always avoids me to recall this thing, I want to forget this bad experience and I don’t want others to know about it.” (P6).

Patients take steps to avoid the reoccurrence of ED, such as avoiding returning to the hospital where the experience of ED occurred and refraining from the use of psychotropic medications.

“I’m embarrassed that this happened, I’m afraid to face these medical staff, I won’t come back to this hospital in order to not have this happen again. “(P14).

Subtheme 3.2 positive coping: Some patients will try to get specifics of the experience by talking to others to rationalize their behavior and be more open about the experience.

“I got them [family members] to give me the whole story, that I was repeating a movement with my hands all the time, and that I should have been planting a seed, I can think of it now. I also saw my old partner. I probably miss that time so much that I did that.” (P10).

“My son told me that I had a urinary catheter and the doctor explained to me that having a urinary catheter meant that I wanted to relieve myself all the time, that’s why I was in such a hurry to run to the toilet.” (P5).

Some patients also sought to identify the causes of postoperative delirium through various means, such as age, anesthetic medications, and disease factors.

“I heard that this (ED) usually occurs in senior patients, and I’m still relatively young, ah, so I’m looking for the reasons why it happened to me, could it be the anesthesia medication, and I’m so afraid that I’m going to fall into that again. situation where I can’t control myself.” (P15).

Analytical interpretation: The divergence in coping strategies highlights patients’ need to reconstruct meaning after an extremely confusional experience. Negative coping reflects avoidance of psychological trauma associated with the loss of autonomy, which may lead to long-term medical fear and avoidance behaviors. In contrast, positive coping represents active cognitive efforts to rationalize bizarre behaviors by seeking medical explanations, reintegrating the chaotic, irrational ED episode into a coherent personal narrative, thereby restoring predictability, a sense of control, and psychological equilibrium.

### Theme 4: outcomes of experiencing ED

Subtheme 4.1 different abilities to recall the experience of ED: The patients’ recollections of the beginning and end of emergence delirium (ED) differed from the actual situation, and there was a partial lack of their own verbal expressions and behaviors. Furthermore, some participants reported that their memories of events occurring during the period of ED were absent.

“I can’t remember where I’ve been after my surgery (scratching my hair)…I have a bit of a vague recollection of being in a hospital bed with my relatives watching over me and being groggy … I went back to sleep. By the time I was fully aware that I was in the hospital it was the next day.” (P2).

“I can’t remember at all what happened then, I just listened to them later on telling me some of the things I was doing, and I tried to think back, and I can’t remember anything.” (P12).

No concept of how long the ED lasted.

“I don’t know how long I was like this (ED), I forget.” (P6).

Some interviewees were concerned about their memory becoming worse and affecting their postoperative cognitive functioning.

“My memory has become very bad, I always lose things, will I be like this afterward oh (sigh).” (P7).

Subtheme 4.2 damaged self-esteem and long-term shame: Some interviewees mentioned that the experience of ED had damaged their self-esteem and undermined the social and family image they had built up.

“I’ve lived to this age, I’m an education professional, and for so many years both in front of my pupils and my family I’ve been very reasonable, but I was at the time when I was completely unable to control my own I always wanted to pull out the tubes on my body, and I couldn’t listen to anyone’s advice, and I even begged them … I feel so ashamed just thinking about it.” (P8).

“I can’t remember exactly what I did, but my daughter described me up to me like a completely unreasonable shrew, yelling at the top of my lungs, and my fellow patients didn’t get a good night’s rest, which made me feel so embarrassed.” (P14).

A very small number of interviewees mentioned that the experience had left them in a permanent state of shame and caused a psychological burden.

“I cared a lot about what people thought of me, my family, friends, and especially my colleagues were there at the time, but this time my image was completely turned upside down, and the thought that I would have to face them in the future, with my face lost, I was always under a lot of pressure, and the psychological pressure was So much (tears).” (P15).

Subtheme 4.3 fear of reoccurrence and fear of the impact on the prognosis of the disease: Some patients mentioned that they still felt a lot of anxiety and fear after the end of ED and were afraid that it would happen again.

“I don’t dare to go to the hospital, I always feel that someone in the hospital is trying to kill me, that feeling of dying is too real, I’m afraid that that experience will happen again.” (P11).

Patients felt that ED delayed their hospitalization and adversely affected their disease prognosis.

“I got better after a long sleep, all my patients who had surgery in the same time frame as me were discharged and I’m still here, I’m getting better a little slower and I’m more worried that this experience is going to make my disease worse.” (P13).

Analytical interpretation: The enduring consequences of ED indicate that the erosion of bodily and cognitive autonomy and the threat to social identity do not dissipate immediately upon the patient’s full recovery of consciousness. Memory gaps and the awareness of one’s own bizarre behaviors—learned only through the accounts of others—create a form of “biographical disruption,” forcing patients to confront altered self-perceptions and generating anticipatory anxiety regarding future medical experiences and overall cancer prognosis.

### Theme 5: the need for healthcare management in the face of ED

Subtheme 5.1 the desire for companionship and psychological support from family members: Patients desire family interaction and companionship while in the PACU in order to seek a sense of familiarity and security.

“After the operation it was as if I had been sent to a place that was not a ward, with my hands tied up, I kept looking for my partner, and the nurses wouldn’t listen to me, and I wished that a family member would stay with me, and talk to me. “ (P3).

“After that experience I had a higher need for family companionship, I didn’t want to face that fear alone.” (P1).

The patient mentioned wanting to seek treatment with a psychologist for positive psychological support.

“I seem to have a disease in my mind, I am under a lot of psychological stress, I would like to see a psychologist for psychological counseling and support.” (P15).

Analytical interpretation: This desire reflects patients’ attachment needs for loved ones and close friends in a vulnerable state. Family members and close friends serve as anchors of reality and sources of safety; their companionship can provide a familiar connection when patients’ self-awareness is fragmented, helping to rebuild a sense of security.

Subtheme 2 weakness of the support system, the desire to receive clear explanations and continued attention: The patient wanted a clear explanation from medical staff about ED.

“What happened during that time was a blank for me. Was my brain damaged? I am not sure. I hope the doctor can give me a clear explanation.” (P12).

The patient was unsure if the experience of ED would have an impact on later life and would like to have ongoing attention from medical staff.

“I see that my fellow patients have been discharged from the hospital and I am a little bit worried if it is a result of this experience, and if this (ED) will have an impact on all of my life in the future, and I would like to have a doctor who keeps an eye on my condition. “ (P13).

Analytical interpretation: Patients’ strong desire for clear explanations and continued medical attention fundamentally stems from the need to reconstruct meaning after a confusional experience and to alleviate threats to identity and health. When patients confront memory gaps and inexplicable physical sensations, they experience extremely high uncertainty. Authoritative and empathetic explanations from healthcare providers not only serve an educational function but also validate patients’ experiences, reassure them that their cognitive autonomy remains intact, and thereby rebuild their trust in the healthcare system and their own bodily integrity.

## Discussion

This study employed a descriptive phenomenological approach to conduct an in-depth exploration of the real experiences and coping strategies of 15 patients who developed ED after radical lung cancer surgery, identifying five core themes: profound perceptual disturbances, intertwined positive and negative emotions, diverse coping strategies, persistent adverse outcomes, and urgent healthcare management needs. The findings reveal the underexplored subjective experiences of patients with ED after radical lung cancer surgery. By addressing the limitations of existing research—which predominantly focuses on quantitative risk factors while neglecting inner experiences—this study provides crucial qualitative evidence for developing patient-centered, comprehensive ED management models.

Patients with ED after radical lung cancer surgery exhibited multidimensional perceptual abnormalities, primarily manifesting as positive and negative audiovisual hallucinations, temporal-spatial and situational disorientation, and behavioral and expressive disorders, which represent the typical manifestations of acute cognitive dysfunction in ED. The content of patients’ hallucinations and disoriented scenarios was closely related to their daily life experiences and past memories, demonstrating significant individualized characteristics. Furthermore, this study included patients with all three ED subtypes: hyperactive, hypoactive, and mixed. Hypoactive ED was more commonly observed in the elderly population; these patients exhibited no obvious agitation but rather presented with drowsiness and delayed responses, making them highly susceptible to missed diagnosis and delayed intervention, which is consistent with previous research (10). Currently, diagnostic criteria for adult ED have not been fully standardized (15), and how to more accurately identify and manage ED remains a key challenge. Existing surveys indicate that a gap persists between clinical attitudes and practices regarding ED management among PACU healthcare teams, with specific practices in routine delirium assessment (standardized scale screening), perioperative ED prevention interventions, and standardized treatment requiring further improvement (16, 17).

This study suggests that PACU healthcare providers may utilize professional scales such as the Nu-DESC and the 4 ‘A’s Test (4AT) (10) as routine screening tools to implement continuous, dynamic assessments throughout the PACU stay for postoperative patients. For hyperactive ED, the PACU healthcare team should consistently maintain a calm demeanor and adopt an acute-phase bedside intervention strategy centered on “acceptance and reassurance,” including gentle reality orientation, continuous environmental reorientation, and cautious use of physical restraint, to ensure patient safety and alleviate their fear. Simultaneously, particular attention should be directed toward the insidious symptoms of hypoactive ED in the elderly population, achieving early identification and early intervention (10), and further exploring the development of multidisciplinary collaborative standardized management protocols or relevant quality assessment indicators to achieve precise identification and multidimensional intervention for ED (18).

In this study, the emotional experiences of patients with ED after lung cancer surgery were complex and diverse, and their abilities to recall the ED experience also varied. The majority of patients had partial absence of their own verbal expressions and behaviors, bore varying degrees of negative emotions and disease uncertainty, worried about the recurrence of delirium, and a small subset of patients remained relatively silent throughout the interview process, trapped in long-term shame and depressive mood, which seriously diminished their quality of life. These findings are largely consistent with the results of Schmitt (19) and Claesson (20) et al. Based on these observations, healthcare providers may employ specific scales to screen patients’ emotional status and psychological problems. For patients experiencing psychological distress following ED, positive psychology interventions such as cognitive behavioral therapy (21), mindfulness-based stress reduction (22), and early psychological intervention (23) may be utilized. In alignment with the trajectory of ED development, healthcare providers should attend to patients’ inner feelings, provide personalized interventions and emotional support, cultivate positive psychological resources to enhance patients’ quality of life, encourage positive self-perception, and ameliorate negative emotions.

In this study, participants’ coping strategies for ED encompassed both negative coping (e.g., avoiding recall, discussion, or returning to the same hospital) and positive coping (e.g., rationalizing their own behavior, actively seeking the causes of ED). The majority of participants perceived the support system as weak and desired clear explanations and continued attention from healthcare providers regarding the occurrence of ED, which was associated with their fear of ED recurrence and concerns about its impact on disease prognosis. Previous research has demonstrated that the occurrence of ED is influenced by multiple factors, including age, sex, pain, and surgical duration (24). For patients undergoing radical lung cancer surgery, prolonged intraoperative one-lung ventilation has also been identified as a contributing factor to ED (25, 26). Therefore, healthcare providers should proactively offer patients explanations regarding the mechanisms of ED, help patients rationalize their abnormal behaviors (e.g., explaining that urinary catheter insertion triggers voiding hallucinations), and guide them toward adopting positive coping strategies. Additionally, given patients’ concerns about disease prognosis and cognitive decline identified in this study, it is necessary to provide positive psychological guidance and sustained attention to their condition, extending the focus of care beyond hospital discharge. Future consideration should be given to developing post-discharge continuity of care models (e.g., leveraging “Internet + Nursing Services,” WeChat mini-programs, or telephone follow-up) to provide home-based patients with professional cognitive function monitoring, psychological counseling, and daily life guidance, thereby meeting their multidimensional needs for continuity of disease management.

Participants in this study also expressed a desire for family interaction and companionship in the PACU to seek a sense of security and familiarity. This may be attributable to the closed and unfamiliar nature of the PACU environment, particularly the significant noise from cardiac monitors and light stimulation. Family involvement is closely associated with the quality of delirium patient care (27). Therefore, it is recommended to optimize the comfort of the PACU environment by reducing auditory and visual stimulation, implementing time-limited family companionship within the scope permitted by institutional policies, or integrating smart nursing technologies with cloud-based family companionship to provide personalized humanistic care, thereby helping patients adapt to the environment, shortening the duration of ED, and enhancing patients’ family and social support.

### Strengths and limitations

This study exhibits notable strengths: it addresses a critical gap in existing literature by adopting a descriptive phenomenological approach to explore the understudied subjective experiences of ED in patients post-radical lung cancer surgery, moving beyond the predominant quantitative focus in ED research to unpack patients’ perceptual disturbances, emotional responses, coping strategies, and unmet needs—insights that are highly relevant for guiding patient-centered clinical practice. Methodologically, it ensures rigor through purposeful sampling (encompassing diverse demographics and ED subtypes) aligned with information saturation principles, systematic data collection (controlled environment, rapport-building, member checking to validate authenticity), and rigorous analysis via the Colaizzi 7-step method with inter-coder consensus to minimize bias. Additionally, the study adheres strictly to ethical standards, with ethics committee approval and informed consent, enhancing its scientific integrity.

However, this study has several limitations. Sample representativeness is constrained by recruitment from a single tertiary hospital in Sichuan Province, excluding patients from other regions or healthcare settings, and the small sample size (n=15) limits generalizability to broader ED populations. Furthermore, memory bias warrants in-depth discussion. As demonstrated by the results of this study, some patients had absent or incomplete memories of the ED episode. From a pathophysiological perspective, the core features of ED involve fluctuating levels of consciousness, impaired attention, and deficits in short-term memory encoding, rendering patients unable to form complete memory traces. At the level of qualitative data analysis, some patients exhibited fragmented and vague memories, some had complete memory absence, and still others could not reconstruct their experiences from their own memories but instead relied on descriptions from family members and healthcare providers to reconstruct the entire process. As this was a retrospective qualitative study, all data were derived from patients’ *post hoc* oral accounts; memory deficits and memory reconstruction objectively exist and may affect the completeness and authenticity of the oral accounts to some extent, which is a limitation difficult to avoid in such research.

Therefore, to address this unavoidable methodological challenge, future qualitative research on delirium experiences should consider employing triangulation, combining patients’ retrospective interviews with real-time clinical observations in the PACU and interviews with family members and nurses, to compensate for the limitations of subjective recall and thereby more comprehensively reconstruct the authentic picture of ED.

## Conclusion

This study provides an in-depth exploration of the real experiences and coping strategies of patients who developed ED after radical lung cancer surgery. The findings reveal that patients perceive the ED experience as a highly complex clinical event characterized by severe perceptual disturbances, temporal/spatial/situational disorientation, loss of bodily and communicative control, profound negative emotions (fear, anxiety, anger, shame, guilt), and concerns about recurrence and prognosis. In response to ED, patients adopted diverse coping strategies, ranging from negative defenses such as avoidance behaviors and maintaining silence to positive attempts including actively seeking medical explanations and narrative reconstruction of abnormal experiences.

Based on these qualitative findings, this study emphasizes that future clinical practice urgently requires the implementation of systematic ED screening to achieve early and precise identification; during ED episodes, acute-phase bedside intervention strategies centered on “acceptance and reassurance” should be implemented; upon patients’ recovery of consciousness, clear communication regarding mechanisms and professional emotional support should be provided to alleviate their sense of stigma; simultaneously, the active involvement of family support systems should be facilitated, and individualized postoperative continuity of care should be initiated to promote comprehensive recovery.

## Data Availability

The original contributions presented in the study are included in the article/supplementary material. Further inquiries can be directed to the corresponding authors.
